# Cortical correlations of CSF Neurogranin, VILIP‐1, Alpha‐Synuclein and NFL in SCD and MCI according to amyloid status

**DOI:** 10.1002/alz.088318

**Published:** 2025-01-09

**Authors:** Emanuel R. Guimarães, Brunno de Campos, Isadora Cristina Ribeiro, Ítalo Karmann Aventurato, Marjorie Cristina Rocha da SIlva, Liara Rizzi, Marcio Balthazar

**Affiliations:** ^1^ Universidade Estadual de Campinas (UNICAMP), Campinas, SP Brazil; ^2^ University of California San Francisco, San Francisco, CA USA

## Abstract

**Background:**

Alzheimer's disease (AD) pathophysiology is complex and not completely known. Emerging new biomarkers that evaluate synaptic function (VILIP‐1, neurogranin), co‐pathology (alpha‐synuclein), and neurodegeneration (NFL) are potential candidates to be incorporated into the early AD diagnosis. To better understand the relevance of these biomarkers, we evaluated the correlations between their CSF concentrations with whole‐brain grey matter volumes in SCD and MCI, according to their amyloid status (A‐ or A+).

**Method:**

75 participants diagnosed with SCD or MCI were included, 30 A‐ and 45 A+. They all underwent comprehensive neuropsychological assessment, CSF analyses (Roche Elecsys), and volumetric 3T MRI (Philips Achieva). Voxel‐based morphometry was used to quantify brain volumes through CAT12 running on MATLAB 2019b. Partial correlation analysis between CSF biomarkers and cortical volumes was performed with SPSS 22, adjusting for age and sex.

**Result:**

Significant correlations were found in the A‐ but not in the A+ group. NFL and hippocampus (r=0.40, p =0.028); VILIP‐1 and superior temporal gyrus (r= ‐0.38, p=0.035), and superior frontal gyrus (r=‐0.40, p=0.025); alpha‐synuclein and hippocampus (r= ‐0.39, p=0.036), middle frontal gyrus (r=‐0.375, p=0.049), and superior temporal gyrus (r=‐0.478, p=0.01).

**Conclusion:**

We found different patterns of correlations between brain anatomy and emerging biomarkers, according to amyloid status, in the very early stage of the neurodegenerative process. Interestingly, these correlations were found only in the amyloid‐negative group, which might suggest that different pathological processes involving synaptic function, neurodegeneration, and co‐pathology occur in suspected non‐Alzheimer pathophysiology cases.

